# Myeloid/Lymphoid Neoplasm with FGFR1::ZMYM2 Rearrangement Presenting as T-Cell Acute Lymphoblastic Lymphoma with Concurrent Myeloproliferative Neoplasm: A Case Report

**DOI:** 10.3390/hematolrep18040056

**Published:** 2026-08-06

**Authors:** Meha Krishnareddigari, Gopal Patel, Aqiba Bokhari, John Paul Graff, Denis M. Dwyre, Arun Panigrahi

**Affiliations:** 1 UCLA Health, University of California, Los Angeles, CA 90025, USA; 2 Department of Pathology and Laboratory Medicine, University of California, Los Angeles, CA 90095, USAabokhari@health.ucdavis.edu (A.B.); jpgraff@health.ucdavis.edu (J.P.G.); dmdwyre@health.ucdavis.edu (D.M.D.)

**Keywords:** FGFR1 rearrangement, ZMYM2, 8p11 myeloproliferative syndrome, T-cell acute lymphoblastic lymphoma, myeloproliferative neoplasm, acute myeloid leukemia, CSF3R, PTEN, hematopoietic stem cell transplantation

## Abstract

**Background:** Myeloid/lymphoid neoplasms with FGFR1 rearrangement (MLN-FGFR1), also known as 8p11 myeloproliferative syndrome, are rare and aggressive hematologic malignancies arising from pluripotent stem cells. The FGFR1::ZMYM2 fusion resulting from t(8;13)(p11.2;q12) characteristically co presents as T-lymphoblastic lymphoma in lymph nodes alongside a myeloproliferative neoplasm in the bone marrow. The disease is resistant to tyrosine kinase inhibitors and conventional chemotherapy, and carries a median survival of less than 12 months without allogeneic hematopoietic stem cell transplantation (allo-HSCT). **Case Presentation**: We report a 23-year-old female who presented with progressive cervical lymphadenopathy and hyperleukocytosis (WBC 186.6 K/μL). Excisional lymph node biopsy demonstrated T-cell acute lymphoblastic lymphoma (T-ALL) with eosinophilic infiltration; immunohistochemistry confirmed lymphoblasts positive for CD1a, CD2, CD3, CD4, CD5, CD7, CD8, and TdT. Concurrent bone marrow biopsy showed a myeloproliferative neoplasm without excess blasts. Chromosomal analysis confirmed t(8;13)(p11.2;q12) with FGFR1::ZMYM2 rearrangement, and NGS identified a concurrent CSF3R variant (Q741*). She received induction chemotherapy per the PEDS AALL1231 protocol (Arm A) followed by consolidation, with a course complicated by hyperleukocytosis, venous thromboembolism, *E. coli* bacteremia, and severe mucositis requiring PICU admission. Despite initial response, the disease progressed to acute myeloid leukemia (AML) with acquisition of a PTEN variant; the patient was offered but did not complete allo-HSCT and died of refractory AML approximately 10 months after diagnosis. **Conclusions**: This case highlights the aggressive clinical course and diagnostic challenges of MLN-FGFR1, a rare stem cell-derived myeloid/lymphoid neoplasm. To our knowledge, this appears to be the first reported case documenting sequential CSF3R and PTEN variant acquisition with complete follow-up through fatal AML transformation, and the first to describe treatment with a pediatric ALL induction protocol (PEDS AALL1231) in this setting. The characteristic histomorphologic pattern of eosinophil-rich T-ALL in lymph nodes with concurrent myeloproliferative neoplasm in bone marrow should prompt immediate molecular workup. Allo-HSCT must be pursued urgently at diagnosis, as complications rapidly narrow the transplant window and the disease is uniformly fatal without it.

## 1. Introduction

Myeloid/lymphoid neoplasms with eosinophilia and rearrangements of PDGFRA, PDGFRB, FGFR1, or PCM1::JAK2 represent a rare and heterogeneous group of hematopoietic malignancies arising from mutated pluripotent stem cells [1]. The clinicopathologic manifestation, treatment response, and prognosis are strongly influenced by the specific rearrangement partner. PDGFRA- and PDGFRB-related diseases typically respond well to imatinib, whereas FGFR1-rearranged disease is uniformly resistant to tyrosine kinase inhibitors and carries a strikingly worse prognosis.

**Figure 1:**
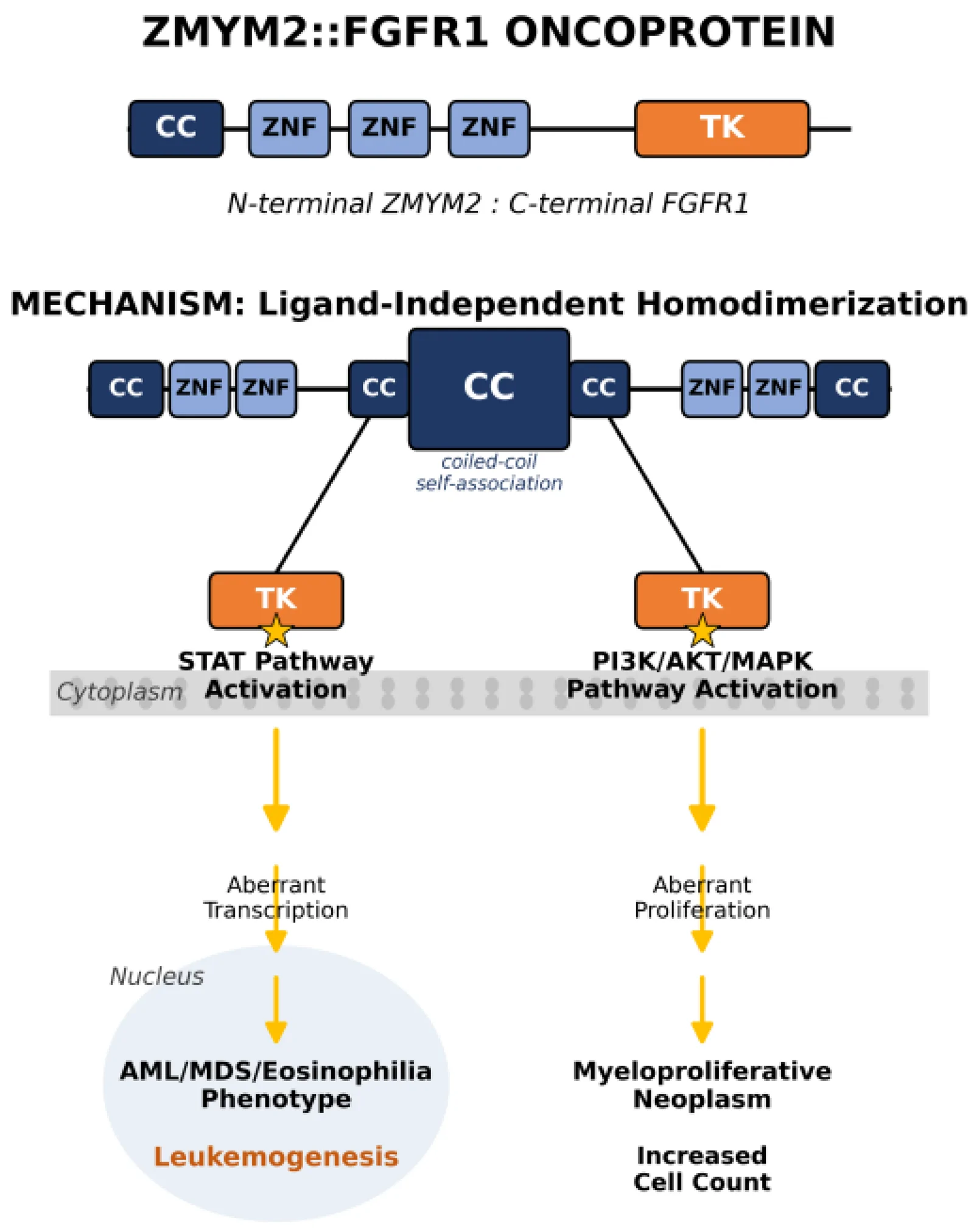
Schematic of the ZMYM2::FGFR1 oncoprotein and proposed mechanism of disease. The fusion retains the N-terminal coiled-coil (CC) and zinc-finger (ZNF) domains of ZMYM2 joined to the C-terminal tyrosine kinase (TK) domain of FGFR1. Self-association through the CC domain drives ligand-independent homodimerization and constitutive TK activation, triggering downstream STAT and PI3K/AKT/MAPK pathway activation. Aberrant transcription downstream of STAT signaling produces the AML/MDS/eosinophilia phenotype and lymphoblastic lymphoma component, while aberrant proliferation downstream of PI3K/AKT/MAPK signaling produces the concurrent myeloproliferative neoplasm, together driving leukemogenesis.

FGFR1-rearranged disease, also known as 8p11 myeloproliferative syndrome, is the most aggressive neoplasm in this category [2]. It results from translocation of the FGFR1 gene at 8p11.2 with one of at least 14 known partner genes. The balanced reciprocal translocation t(8;13)(p11.2;q12) involving FGFR1 and ZMYM2 at 13q12.1 is the most frequently observed genetic abnormality in this subgroup. As illustrated in Figure 1, the resulting ZMYM2::FGFR1 fusion protein retains the N-terminal coiled-coil and zinc-finger domains of ZMYM2 joined to the C-terminal tyrosine kinase domain of FGFR1; self-association through the ZMYM2 coiled-coil domain drives ligand-independent homodimerization and constitutive tyrosine kinase activation, triggering downstream STAT and PI3K/AKT/MAPK signaling that produces the characteristic dual lymphoid and myeloid phenotype of this disease. This translocation characteristically manifests as concurrent T-cell lymphoblastic lymphoma with eosinophilia in lymph nodes alongside a myeloproliferative neoplasm in a hypercellular bone marrow.

Co-occurring somatic mutations are identified in approximately 70–80% of MLN-FGFR1 cases, with RUNX1 mutations representing the most frequent co-alteration, occurring in up to 80% of mutated cases and constituting the only recurrently mutated gene identified in the FGFR1-rearranged subgroup of larger cohorts; other reported co-mutations include those affecting ASXL1, TET2, and rarely CSF3R [2,3].

The disease affects a wide age range (3–84 years) with a mean onset of approximately 32 years. Patients typically present with systemic symptoms, lymphadenopathy, and organomegaly. Cases presenting initially in chronic phase frequently transform to acute leukemia. Despite high expression of aberrant tyrosine kinase activity, MLN-FGFR1 does not respond to conventional tyrosine kinase inhibitors, is highly resistant to standard chemotherapy, and carries a median survival of less than 12 months [4]. Allo-HSCT represents the only potentially curative option and should be pursued in all eligible patients.

Here we describe the clinical course of a 23-year-old female with FGFR1::ZMYM2 rearrangement who presented with cervical lymphadenopathy, received induction and consolidation chemotherapy complicated by multiple life-threatening events, and ultimately died of refractory AML approximately 10 months after diagnosis, consistent with the grim prognosis reported in the literature. This case is presented to raise awareness of this rare entity and the critical importance of early transplant referral.

## 2. Case Report

### 2.1. Initial Presentation

A 23-year-old female presented with a two-week history of progressively worsening cervical lymphadenopathy. She had initially been evaluated at an outside hospital emergency department where a head CT demonstrated lymphadenopathy without abscess. The swelling progressed to the point of dysphagia for liquids, prompting transfer to an outside community hospital, where a repeat head CT confirmed lymphadenopathy and complete blood count revealed a white blood cell count of 186.6 K/μL. She was subsequently transferred to our institution for further management.

On admission, laboratory evaluation demonstrated markedly elevated white blood cell count at 103.4 K/μL with left-shifted granulocytosis, consistent with hyperleukocytosis. Physical examination was notable for bilateral cervical lymphadenopathy.

### 2.2. Pathologic Findings

**Figure 2:**
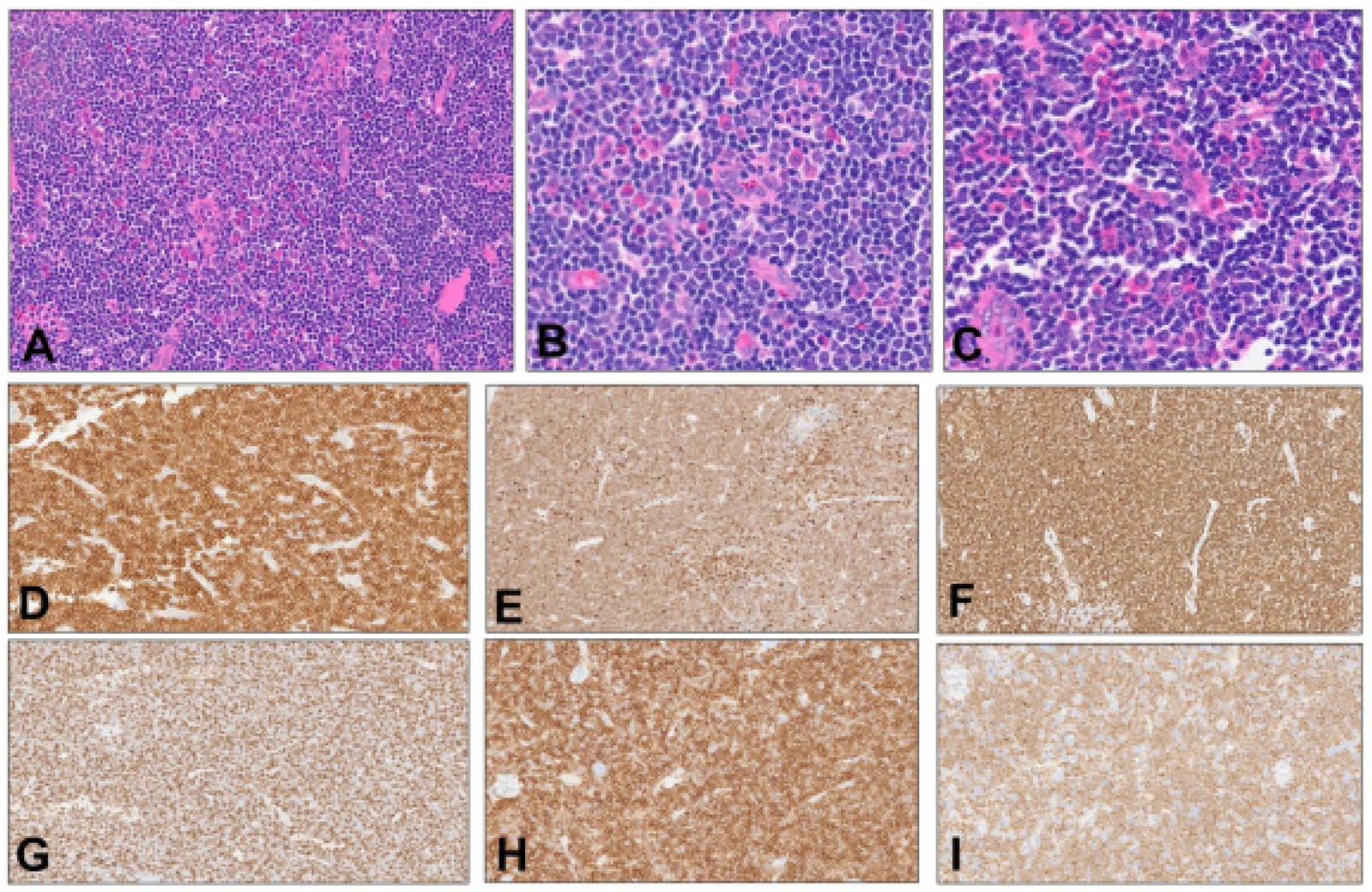
Histopathologic examination of the right cervical lymph node, consistent with T-cell lymphoblastic lymphoma (T-LBL), demonstrating complete effacement of nodal architecture by diffuse sheets of infiltrating blasts with admixed eosinophils dispersed throughout the excisional biopsy; scattered high-endothelial venules are present. Three representative fields: (**A**) H&E stain, 10× magnification; (**B**,**C**) H&E stain, 40× magnification. Immunohistochemical profile of the blasts: (**D**) CD3 positive (cytoplasmic, with a perinuclear dot-like pattern), 20×; (**E**) CD7 positive, 20×; (**F**,**G**) CD4 positive/CD8 positive (double positive), 20×; (**H**) CD1a positive, 20×; (**I**) TdT positive, 20×. Ki67 was approximately 70%; CD5 and CD2 were positive.

Excisional biopsy of the right cervical lymph node demonstrated completely effaced nodal architecture, with a brisk hematolymphoid infiltrate comprised predominantly of lymphoblasts and admixed eosinophils scattered throughout the specimen (Figure 2A–C). Immunohistochemical analysis showed the blasts to be diffusely and strongly positive for CD3 (cytoplasmic, with a perinuclear dot-like pattern), CD2, CD5, CD7, CD1a, and TdT, and to be double-positive for CD4 and CD8 (Figure 2D–I). The proliferation index (Ki67) was approximately 70%. The blasts were negative for CD20, CD10, CD57, ALK1, and EBER; CD34 immunostaining was not performed. The combined morphology and immunophenotype were consistent with T-cell lymphoblastic lymphoma (T-LBL). Concurrent bone marrow biopsy revealed a hypercellular marrow with myeloid-predominant maturation and no increase in blasts, consistent with a myeloproliferative neoplasm (MPN).

From a diagnostic standpoint, it was this combined morphologic and immunophenotypic pattern—a T lymphoblastic infiltrate with prominent background eosinophilia in the lymph node, occurring concurrently with an otherwise unexplained myeloproliferative process in the bone marrow—that raised early suspicion for an underlying FGFR1 rearrangement and prompted the cytogenetic workup described below. We highlight this pattern explicitly as a teaching point: this dual-compartment presentation should prompt hematopathologists and hematologists to pursue FGFR1 cytogenetic and molecular studies even before rearrangement is confirmed, given the diagnostic and prognostic implications of an early versus delayed diagnosis.

BCR-ABL gene rearrangement was not detected on the MPN FISH panel. Subsequent chromosomal analysis demonstrated t(8;13)(p11.2;q12) involving the FGFR1 and ZMYM2 genes, confirming the diagnosis of myeloid/lymphoid neoplasm with FGFR1 rearrangement. NGS additionally identified a CSF3R variant (Q741* NM_156039.3:c.340C>T).

### 2.3. Treatment Course

**Figure 3:**
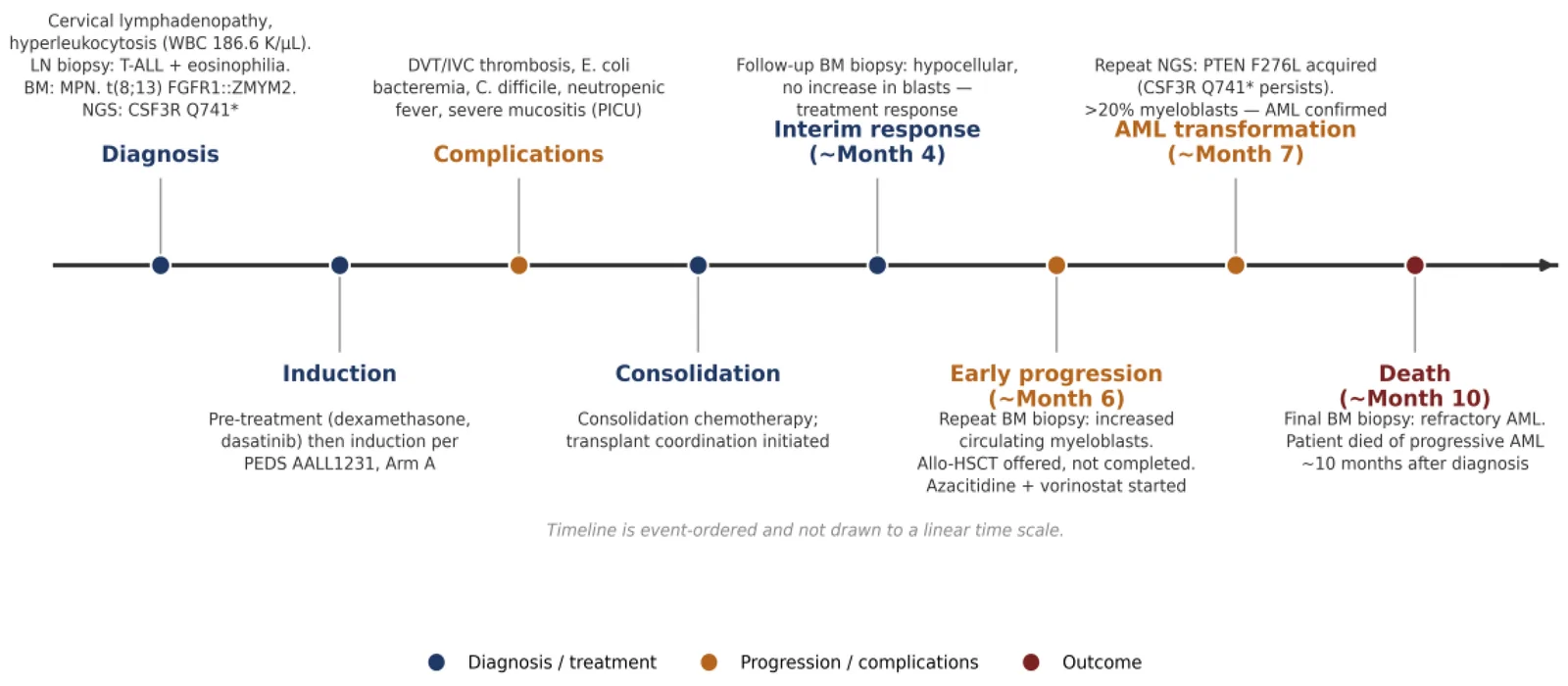
Timeline of major clinical, pathologic, and molecular events from diagnosis to death. Events are ordered chronologically using relative timepoints as described in the manuscript and are not drawn to a linear time scale.

The overall clinical, pathologic, and molecular timeline from diagnosis to death is summarized in Figure 3.

Following confirmation of T-ALL with concurrent MPN in the setting of FGFR1::ZMYM2 rearrangement, the patient was initiated on pre-treatment with dexamethasone 9 mg twice daily and dasatinib 140 mg daily. Induction chemotherapy was administered per the PEDS AALL1231 protocol, Arm A, consisting of intrathecal cytarabine (under fluoroscopy), dexamethasone 15 mg twice daily, vincristine on Days 1 and 8, daunorubicin on Days 1 and 8, pegasparaginase on Day 4, and intrathecal methotrexate.

The patient was admitted for a prolonged inpatient course requiring intensive multidisciplinary management. Consolidation chemotherapy included vincristine and intrathecal methotrexate, 6-mercaptopurine (Days 29–42), cytarabine, and intermediate-dose methotrexate with leucovorin rescue. Chemotherapy was modified due to severe peripheral neuropathy that precluded use of ponatinib.

The inpatient course was complicated by multiple serious adverse events. From a hematologic standpoint, she developed profound chemotherapy-induced pancytopenia requiring multiple transfusions of packed red blood cells and platelets, and received filgrastim. She developed right upper extremity deep vein thrombosis with subsequent suprarenal inferior vena cava thrombosis identified on duplex ultrasound, managed with anticoagulation and supportive therapy; rivaroxaban was initiated but held for the majority of admission given platelets below 50 K/μL. Infectious complications included *E. coli* bacteremia identified on blood culture, treated with cefepime and tobramycin with subsequent transition to ceftriaxone per susceptibilities for a total seven-day course, as well as a C. difficile infection managed with oral vancomycin. She redeveloped neutropenic fever and was restarted on cefepime (continued through ANC recovery) and vancomycin, with micafungin and Bactrim/pentamidine for prophylaxis. She developed severe mucositis causing inability to take oral medications and severe pain, requiring a brief PICU admission for respiratory monitoring while on morphine PCA given her underlying obstructive sleep apnea. Cardiovascular management included scheduled intravenous labetalol and hydralazine PRN during the period she was unable to take oral antihypertensives. She also developed significant neuropathic foot pain managed with a multimodal regimen including gabapentin, amitriptyline, ketamine, and opioid analgesics.

Upon discharge, transplant coordination had been initiated, with pulmonary function testing and dental evaluation completed in anticipation of allo-HSCT.

### 2.4. Disease Progression and Outcome

Follow-up bone marrow biopsy performed four months after the initial biopsy showed a hypocellular marrow with myeloid-predominant hematopoiesis and no increase in myeloblasts or lymphoblasts, indicating a treatment response. However, two months later, repeat bone marrow biopsy and peripheral blood evaluation revealed increased and circulating myeloblasts, indicating early disease progression.

At this time, the patient was offered allogeneic hematopoietic stem cell transplantation but did not complete the procedure. She subsequently received MDS-directed therapy with azacitidine and vorinostat following AML progression. Repeat bone marrow biopsy demonstrated progression to AML with FGFR1 rearrangement, defined by greater than 20% myeloblasts. Repeat NGS confirmed the previously identified CSF3R variant and additionally identified a PTEN variant (F276L NM_000314.8:c.834C>G), consistent with molecular disease progression.

The final bone marrow biopsy, performed in the weeks preceding death, demonstrated a hypercellular marrow with markedly increased myeloblasts exceeding 20% of marrow cellularity, confirming transformation to AML with FGFR1 rearrangement. The marrow showed dysplastic changes across multiple lineages with near-complete replacement of normal hematopoiesis by blast forms. Peripheral blood flow cytometry at this time demonstrated approximately 40% circulating abnormal myeloblasts consistent with involvement by acute myeloid leukemia, with no abnormal T-lymphoblasts noted. The myeloblast immunophenotype was dim CD45-positive with low side scatter, CD34-positive, a dim subset CD117-positive, HLA-DR-positive, dim CD11c-positive, CD33-positive, dim CD13-positive, CD38-positive, a subset CD56-positive, and dim CD7-positive; the myeloblasts were negative for CD3, CD2, CD5, CD8, CD4, CD14, CD15, CD16, kappa, lambda, TdT, CD138, myeloperoxidase, CD22, CD79a, CD19, and CD20. Cytogenetic analysis confirmed persistence of t(8;13)(p11.2;q12) without acquisition of additional cytogenetic abnormalities. Repeat NGS studies confirmed the previously identified CSF3R (Q741*) and PTEN (F276L) variants, with no new actionable mutations identified. The overall findings were consistent with refractory AML arising in the setting of MLN-FGFR1, with molecular evidence of clonal evolution through sequential mutational acquisition.

The patient died of refractory, rapidly progressive AML approximately 10 months after initial diagnosis. Her family was present at the time of her death. The overall clinical course from the initial presentation with hyperleukocytosis and T-ALL, through a treatment-refractory myeloproliferative phase, to AML transformation and death within less than 12 months is consistent with the natural history of FGFR1::ZMYM2-rearranged myeloid/lymphoid neoplasm as reported in the literature.

## 3. Discussion

We report a case of MLN-FGFR1 with ZMYM2 rearrangement in a 23-year-old woman whose clinical course exemplifies the characteristic presentation and uniformly poor prognosis of this rare entity. To our knowledge, based on a comprehensive review of the available literature, this case is notable for three reasons not previously reported in combination in the MLN-FGFR1 literature: (1) sequential acquisition of CSF3R and PTEN variants with complete molecular documentation through fatal AML transformation; (2) treatment with a pediatric ALL induction protocol (PEDS AALL1231, Arm A), providing novel real-world therapeutic data in a disease without an established chemotherapy standard; and (3) a fully documented fatal clinical course in a young patient, from initial presentation to death within 10 months, consistent with the reported median survival of less than 12 months. Together, these features advance the clinical understanding of this disease beyond what has been previously described. In particular, this case adds to the small existing body of MLN-FGFR1 case series by illustrating in molecular detail how clonal evolution can unfold over the disease course, and by underscoring how rapidly the window for curative allo-HSCT can close once life-threatening complications arise—reinforcing that transplant timing, and not treatment intensity alone, may be a decisive factor in outcome [4].

The initial presentation of this case, including marked hyperleukocytosis, bilateral cervical lymphadenopathy, and the histomorphologic finding of T-LBL with prominent eosinophilic infiltration in the lymph node concurrent with a myeloproliferative neoplasm in the bone marrow, is the hallmark of FGFR1::ZMYM2 rearrangement. The full lymphoblast immunophenotype in this case, positive for CD1a, CD2, cytoplasmic CD3, CD4/CD8 (double positive), CD5, CD7, and TdT (Figure 2), is characteristic of T lymphoblastic lymphoma and, together with the dual-compartment pattern of T-lymphoproliferative disease in lymph nodes with myeloproliferative disease in bone marrow, is well-established in the literature and should immediately prompt fluorescence in situ hybridization (FISH) and/or chromosomal analysis for FGFR1 rearrangement [1,5].

Mechanistically, the ZMYM2::FGFR1 fusion protein drives disease through ligand-independent homodimerization (Figure 1). Retention of the ZMYM2 coiled-coil domain enables constitutive self-association of the fusion protein independent of any extracellular ligand, juxtaposing two FGFR1 tyrosine kinase domains and triggering constitutive trans-autophosphorylation. This aberrant kinase activation drives both STAT pathway signaling, promoting the aberrant transcriptional program underlying the lymphoblastic and eosinophilic phenotype, and PI3K/AKT/MAPK pathway signaling, promoting the aberrant proliferation underlying the concurrent myeloproliferative neoplasm. This dual downstream signaling elegantly explains the simultaneous lymphoid and myeloid phenotypes observed in a single patient, as in the case presented here.

The molecular findings in this case are particularly noteworthy and warrant careful mechanistic interpretation. The concurrent CSF3R Q741* variant identified at diagnosis is a nonsense mutation causing truncation of the cytoplasmic domain of the granulocyte colony-stimulating factor receptor. This is mechanistically distinct from CSF3R T618I, a membrane-proximal ‘type 1’ activating mutation and the canonical diagnostic mutation for chronic neutrophilic leukemia (CNL) and some cases of atypical chronic myeloid leukemia [6,7]. Truncating ‘type 2’ mutations such as Q741* instead cause loss of the C-terminal internalization domain, resulting in sustained receptor surface expression and impaired receptor downregulation; while not activating in the conventional CNL sense, this altered receptor trafficking may still prolong downstream signaling and could plausibly contribute to the pronounced neutrophilia and leukocytosis observed at presentation [6,7]. Importantly, CNL diagnosis requires a CSF3R T618I or other membrane-proximal activating mutation; the Q741* truncation does not meet this criterion, and the dominant molecular driver in this case is unambiguously the FGFR1::ZMYM2 fusion, making a separate CNL diagnosis both mechanistically unsupported and diagnostically inappropriate here.

The subsequent acquisition of a PTEN F276L variant at the time of AML transformation may have contributed to disease progression, as PTEN loss is known to activate the PI3K/AKT/mTOR pathway, promote blast survival and proliferation, and associate with leukemic transformation and chemotherapy resistance in several myeloid malignancies. We present this interpretation as hypothesis-generating rather than definitive, given that a single-patient case report cannot establish causality between the PTEN variant and the observed transformation. Nonetheless, the sequential accumulation of these variants illustrates the dynamic mutational landscape of MLN-FGFR1 and reinforces that disease progression is not solely driven by the founding FGFR1 fusion but by ongoing genomic instability.

This raises a biologically important question: does this case represent a single clonal disease in which FGFR1::ZMYM2, CSF3R Q741*, and later PTEN F276L all arose within one evolving malignant clone across different anatomic compartments, or could it instead reflect two independent, biclonal neoplasms coincidentally co-occurring in the same patient? True biclonal hematologic disease is rare but biologically plausible and has not, to our knowledge, been systematically excluded in prior MLN-FGFR1 reports. Definitive clonality determination (for example, variant allele fraction tracking of the fusion transcript and point mutations together, or single-cell sequencing) was not performed in this case, which we acknowledge as a limitation. However, precedent from larger genomic cohorts of FGFR1-rearranged disease, in which co-occurring mutations such as RUNX1 have been shown to be subclonal at diagnosis and to expand within the FGFR1-rearranged clone over time, supports a model of clonal evolution within a single disease rather than biclonal neoplasia [2]. The sequential appearance of CSF3R Q741* at diagnosis followed by PTEN F276L at AML transformation, both in the persistent context of the same FGFR1::ZMYM2 rearrangement without acquisition of a second independent cytogenetic clone, is most consistent with this single-clone model, though we agree with the need for correlative VAF and clonality data in future cases to confirm this definitively.

The treatment course in this case reflects the profound challenges of managing MLN-FGFR1. Despite aggressive induction therapy per a pediatric ALL protocol (PEDS AALL1231, Arm A), a reasonable approach given the dominant T-ALL presentation, the disease ultimately progressed. The addition of dasatinib as a pre-treatment agent, although not a standard FGFR1 inhibitor, may have been used empirically given the co-existing MPN features; however, MLN-FGFR1 is well-documented to be resistant to available tyrosine kinase inhibitors [1,4]. The multiple life-threatening complications encountered, including hyperleukocytosis, IVC thrombosis, bacteremia, and severe mucositis requiring PICU admission, underscore the clinical fragility of these patients and the narrow window for transplant preparation.

Most cases of MLN-FGFR1 rapidly transform due to concurrent or acquired additional genetic mutations and chromosomal abnormalities [5]. Rare cases with KIT-positive systemic mastocytosis [8], bilineal [9], or trilineal [5,10] lymphomas have also been reported, further illustrating the pluripotent stem cell origin of this disease. The morphology of the excised lymph node, increased eosinophils infiltrating concurrently with T acute lymphoblastic lymphoma, is a distinctive and diagnostically important pattern in FGFR1::ZMYM2-translocated disease.

The fatal outcome in this case, in which the patient was offered but did not complete allo-HSCT, reinforces the existing literature that transplantation is the only potentially curative option. In a retrospective analysis by Strati et al., patients who underwent allo-HSCT had significantly improved outcomes compared to those who did not [3]. The identification of MLN-FGFR1 should be treated as a transplant emergency, with immediate referral and expedited donor search initiated at diagnosis.

Novel FGFR inhibitors, including pemigatinib and erdafitinib, have shown early promise in FGFR-rearranged malignancies more broadly, though the current supporting evidence in MLN-FGFR1 specifically is limited to case reports and early-phase trials rather than controlled prospective data. Given this limited evidence base, we view these agents as potential adjuncts to help bridge a patient to allo-HSCT rather than as a substitute for transplantation, which remains the only therapy of demonstrated curative potential in this disease. This case adds to the growing body of evidence supporting rapid molecular diagnosis, early transplant referral, and the need for prospective clinical trials evaluating FGFR-targeted therapy in this disease. Given the rarity of MLN-FGFR1, we believe such trials, along with collaborative multi-institutional registries and pooled case series, will likely be necessary to generate the statistical power needed to define optimal induction and bridging strategies and to clarify the true benefit of FGFR-targeted agents in this population.

### Limitations

This report has several limitations inherent to single-patient case reports. As a single case, it cannot establish causality between the acquired molecular variants (CSF3R Q741*, PTEN F276L) and the observed clinical and pathologic transitions, and our interpretations regarding their contribution to disease biology should be regarded as hypothesis-generating. Functional assays to confirm the biological activity of these variants were not performed. Definitive clonality analysis distinguishing single-clone evolution from biclonal disease, such as variant allele fraction tracking across compartments or single-cell sequencing, was also not available. Despite these limitations, we believe the value of this report lies in its detailed, longitudinal clinical and molecular documentation of a rare and poorly characterized entity, which remains scarce in the existing literature.

## 4. Conclusions

We present a case of MLN-FGFR1 with ZMYM2 rearrangement in a young woman whose clinical course, from initial presentation with hyperleukocytosis and dual-compartment T-ALL/MPN, through treatment refractory AML transformation with sequential CSF3R and PTEN mutations, to death within 10 months of diagnosis, encapsulates the aggressive natural history of this disease. The characteristic histomorphologic presentation of eosinophil-rich T-lymphoblastic lymphoma in lymph nodes with concurrent myeloproliferative neoplasm in bone marrow should prompt immediate chromosomal analysis and NGS.

Allo-HSCT must be pursued urgently, as it represents the only potentially curative option. This case is dedicated to our patient, whose brief life and difficult illness motivate efforts to improve recognition and treatment of this rare and fatal disease.

## Data Availability

The data supporting the findings of this case report are contained within the manuscript. Further de identified data are available from the corresponding author upon reasonable request, subject to applicable privacy and institutional regulations.
